# Applications designed to successfully implant in challenging left atrial appendage occlusion cases: a new tool for the interventional cardiologist

**DOI:** 10.1007/s10554-021-02250-y

**Published:** 2021-05-06

**Authors:** José Ramón López-Mínguez, Ginés Martínez-Cáceres, Reyes González-Fernández, Juan Manuel Nogales-Asensio, Victoria Millán-Núñez

**Affiliations:** 1 Interventional Cardiology Section, Cardiology Service, University Hospital of Badajoz, Avda Elvas sn Badajoz, 06080 Badajoz, Spain; 2 Image Cardiology Section, Cardiology Service, University Hospital of Badajoz, Badajoz, Spain

**Keywords:** Left atrial appendage, On CT-scan and image engineering, FEops application

## Abstract

An 85-year-old patient with permanent atrial fibrillation with a DDD pacemaker, and with indication for left atrial appendage occlusion (LAAO). Sent for LAAO due to recurrent gastrointestinal bleedings even on apixaban and with a CHA 2 DS 2 VASc and HAS-BLED scores of 4 and 3 respectively.

An 85-year-old patient with permanent atrial fibrillation with a DDD pacemaker, and with indication for left atrial appendage occlusion (LAAO). Sent for LAAO due to recurrent gastrointestinal bleedings even on apixaban and with a CHA_2_DS_2_VASc and HAS-BLED scores of 4 and 3 respectively.

The TEE (transesophageal echo) showed a chicken wing appendage with a very wide ostium and a very short landing zone due to an extreme angulation (Fig. 1a1 and a2). The landing zone measurements were around 24 mm. These same data are objectified in the angiography with measurements very close to those measured with echo. No angiography projections were adequate found in order to visualize the appropriate landing zone. CT-scan was not initially considered due to moderate chronic kidney disease. A 28 mm Amplatzer™ Amulet™ device was selected which, although angiographically seemed to be anchored, did not provide the safety guarantees or adequate coaxiality in the TEE (Fig. 1b1, b2, C). Thus, it was decided not to implant and suspend the procedure. Then, a CT-scan with a 3-D reconstruction was performed (Fig. 2, top row), showing a type III left atrial appendage and a FEops analysis was requested (Fig. 2, bottom row). With the simulation support of FEops HEARTguide™, a 34 mm Amplatzer™ Amulet™ device was implanted proximally achieving a good result after a laborious procedure (Fig. 1d, E1, E2, F). At 3-month follow-up, the patient is clinically stable, and the good result of the device persists.

**Fig. 1:**
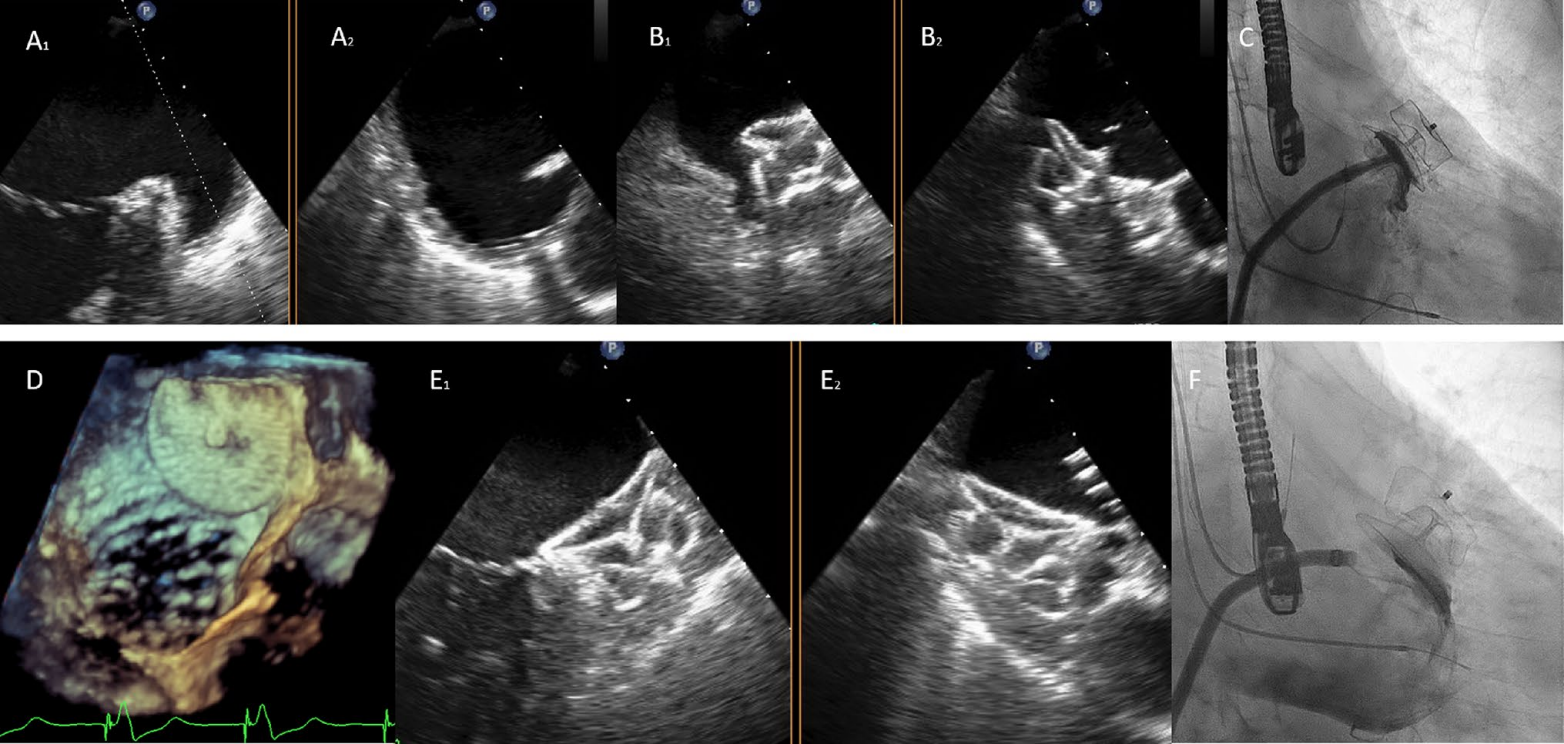
**a1** and **a2** Transesophageal echocardiography (TEE) of the left atrial appendage in X-plane view (65/-24). **b1** and **b2**: the image shows a non-appropriate orientation and apposition with the 28 mm AMULET device. **c** Angiography showing a lack of compression on the device and although there was no contrast inside it was decided not to release the device. With the use of FEops application a 34 mm Amulet device was implanted and released after checking good apposition and compression. **d** tridimensional TEE image of the device. **e1**, **e2** and **f** TEE and angiographic images showing correct colocation of the Amulet device

**Fig. 2:**
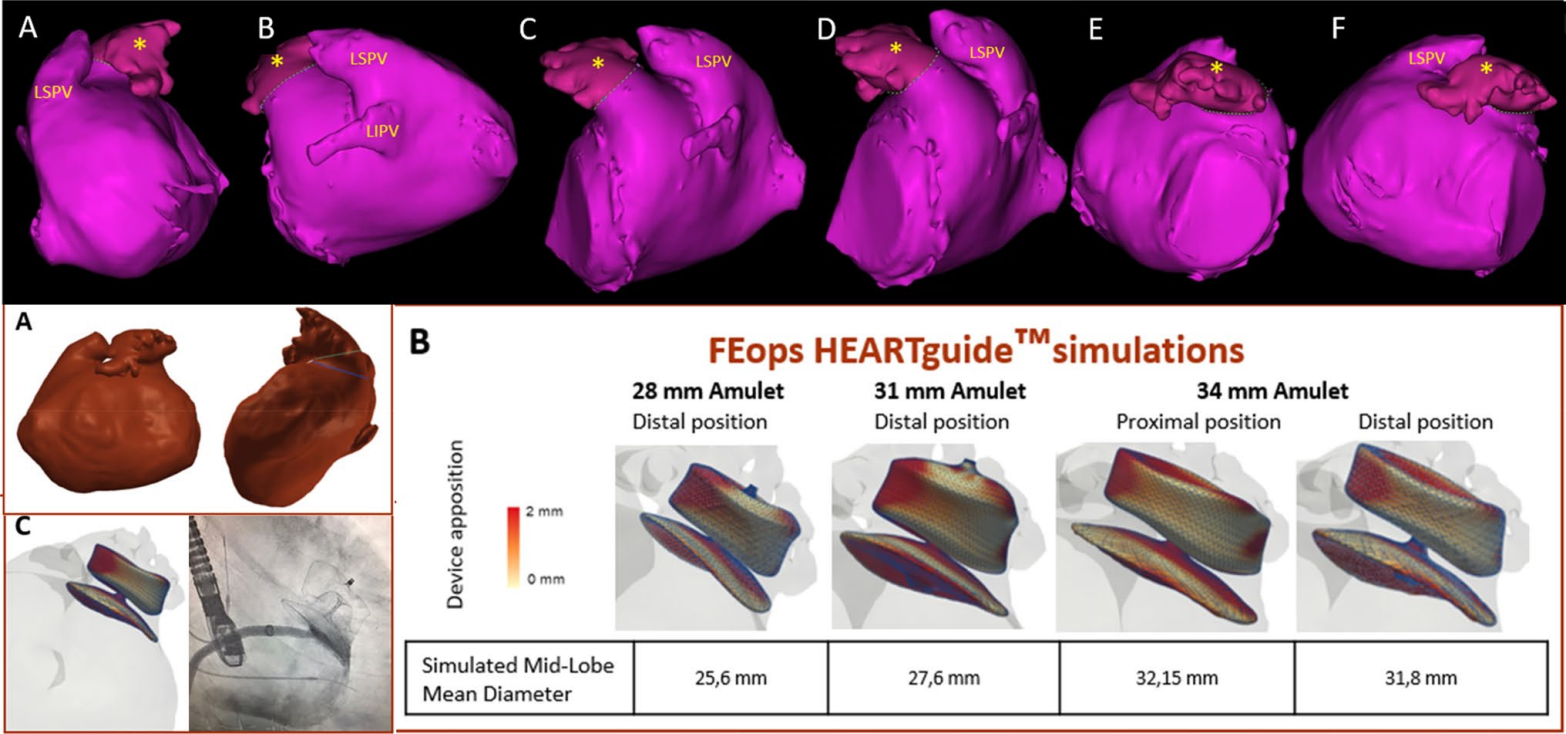
Top row: Superior CT reconstruction of the left atrial appendage (LAA) (*), seen progressively rotating from extreme posterior (**a**–**f**) to anterior projection counterclockwise. LSPV-left superior pulmonary vein. LIPV-left inferior pulmonary vein. Bottom row. Analysis received from FEops application. **a** Left atrial and LAA seen in anterior and lateral LAA projections. Blue lines: ostium and landing zone measurements of LAA. **b** Simulations with 3 sizes of Amplatzer™ Amulet™ devices 28, 31 and 34 mm. From this last one, proximal or distal implant and apposition degree are also simulated. **c** Size (34 mm) and shape of the chosen implant (distal) and the result of the real implant in the procedure

## Discussion

The percentage of successful device implantation (procedural success) for LAAO ranges from 96 to 98% in the most current records. The unfavorable anatomy of some left atrial appendages (LAAs), means that even in the hands of expert operators there may be a 2 to 3% of cases where either the device cannot be implanted (technical failure), or it is not implanted in the most effective way (procedural failure) [1]. LAAs with a very short landing zone, are typical cases of extreme complexity due to a very early lobulation or a very early turn (chicken wing) [2]. There is another type of appendage, reversed chicken wing, whose true complexity lies in its very low and posterior appendage with an ostium that comes out not below the LSPV or between both PVs, but of the LIPV and that it is described as type III in a previous classification (Fig. 2) [3]. It usually coexists with a very wide neck in a constant curve from the beginning towards anterior and superior (a reverse chicken wing morphology), which hardly leaves an area for the correct device anchorage. In addition, it is especially complex to find an adequate working projection in both the echocardiogram and in the angiography [3].

In complex cases such as the one presented, it is recommended when there is no certainty of an optimal result, to suspend the implant and resort to applications such as FEops. This type of simulation is based on CT-scan and image engineering to choose the working projection, the device size and its degree of compression depending on the depth of the landing zone and the size selected (Fig. 2) [4].
